# The dwarf neon rainbowfish *Melanotaenia praecox*, a small spiny‐rayed fish with potential as a new Acanthomorpha model fish: II. Establishment of a microinjection procedure for genetic engineering

**DOI:** 10.1002/dvdy.698

**Published:** 2024-02-05

**Authors:** Kazuhide Miyamoto, Gembu Abe, Koichi Kawakami, Koji Tamura, Satoshi Ansai

**Affiliations:** ^1^ Laboratory of Organ Morphogenesis, Department of Ecological Developmental Adaptability Life Sciences, Graduate School of Life Sciences Tohoku University Sendai Japan; ^2^ Division of Developmental Biology, Department of Functional Morphology, School of Life Science Faculty of Medicine, Tottori University Yonago Japan; ^3^ Laboratory of Molecular and Developmental Biology National Institute of Genetics Shizuoka Japan; ^4^ Department of Genetics The Graduate University for Advanced Studies Shizuoka Japan; ^5^ Laboratory of Molecular Ethology, Department of Integrative Life Sciences Graduate School of Life Sciences, Tohoku University Sendai Japan; ^6^ Present address: Laboratory of Genome Editing Breeding, Graduate School of Agriculture Kyoto University Kyoto Japan

**Keywords:** atheriniformes, CRISPR, Melanotaeniidae, *Tol2* transgenesis, transgenesis methodology, tyrosinase

## Abstract

**Background:**

Rainbowfish is a clade of colorful freshwater fish. *Melanotaenia praecox* is a small rainbowfish species with biological characteristics that make it potentially useful as an experimental model species. We anticipate that *M. praecox* could become a new model used in various fields, such as ecology, evolution, and developmental biology. However, few previous studies have described experimental set‐ups needed to understand the molecular and genetic mechanisms within this species.

**Results:**

We describe detailed procedures for genetic engineering in the rainbowfish *M. praecox*. By using these procedures, we successfully demonstrated CRISPR/Cas‐mediated knockout and *Tol2* transposon‐mediated transgenesis in this species. Regarding the CRISPR/Cas system, we disrupted the *tyrosinase* gene and then showed that injected embryos lacked pigmentation over much of their body. We also demonstrated that a *Tol2* construct, including a GFP gene driven by a ubiquitous promoter, was efficiently integrated into the genome of *M. praecox* embryos.

**Conclusions:**

The establishment of procedures for genetic engineering in *M. praecox* enables investigation of the genetic mechanisms behind a broad range of biological phenomena in this species. Thus, we suggest that *M. praecox* can be used as a new model species in various experimental biology fields.

## Key Findings

- We describe detailed procedures for genetic engineering in the rainbowfish Melanotaenia praecox.
- We successfully demonstrated CRISPR/Cas‐ediated knockout and Tol2 transposon‐mediated transgenesis in this species.
- The establishment of procedures for genetic engineering in M. praecox enables investigation of the genetic mechanisms behind a broad range of biological phenomena in this species.

## 1 INTRODUCTION

**FIGURE 1:**
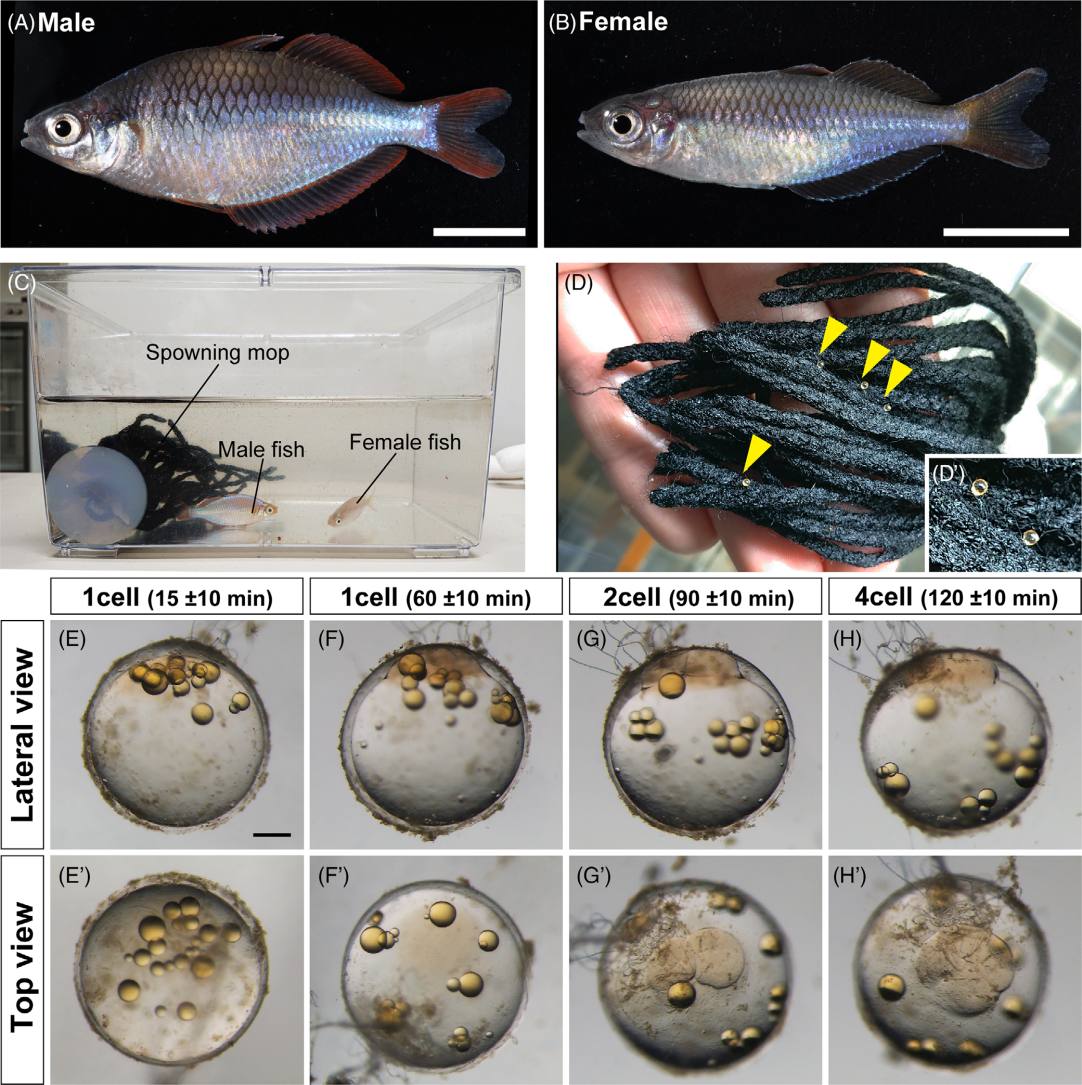
Collection of fertilized eggs in *Melanotaenia praecox*. (A), (B) Representative images of adult males (A) and females (B). (C) Each mature male and female pair was kept in a 3 L breeding tank containing a spawning mop. (D), (D’) Eggs attached to the spawning mop by their attaching filaments. A magnified view of the attached eggs (D’). Yellow arrowheads indicate the attached eggs. (E)–(H’) Progress in the first and second cleavages of an *M. praecox* egg at the one‐cell stage, 15 ± 10 (E), (E’) and 60 ± 10 min (F), (F’) after fertilization; the two‐cell stage at 90 ± 10 min (G), (G’); and the four‐cell stage at 120 ± 10 min (H), (H’). Scale bar indicates 1 cm (A), (B) and 200 μm (E).

Rainbowfish is a clade of colorful freshwater fish that is widely reared in aquaria around the world. Rainbowfish species are distributed across Australia, New Guinea, and their surrounding islands. Rainbowfish is highly divergent in its morphology and coloration^1^, ^2^, ^3^ and exhibits impressive adaptive radiation.^4^, ^5^, ^6^ Rainbowfish (also known as the Melanotaeniidae family) consists of 10 currently recognized genera and belongs to the order Atheriniformes.^2^, ^7^ The genus *Melanotaenia*, the largest genus in Melanotaeniidae,^2^, ^8^ includes *Melanotaenia praecox*, a small freshwater fish species widely bred and raised by hobbyists (Figure 1A, B). According to a previous description^9^ and our own observations, this species has biological characteristics that make it potentially valuable as an experimental model species, such as its small size (~5 cm in adult fish), short generation time (5 months after hatching), and egg spawning all year round.^10^ These characteristics enable us to maintain and breed this species easily in aquarium systems similar to those used for zebrafish and medaka. Furthermore, although scarce genomic information on *M. praecox* is available, genomic data for the closely related species *M. boesemani* are available and helpful. *M. praecox* also has certain characteristics of value in the fields of ecology, evolution, and developmental biology. For example, it has spiny rays in its dorsal, anal, pectoral, and pelvic fins.^11^, ^12^ Spiny rays are regarded as one of the innovations of Acanthomorpha, to which rainbowfish belong, whose diversity and functional significance have fascinated many biologists.^13^, ^14^, ^15^ Although we expect that *M. praecox* could become a new model fish in experimental biology, to our knowledge no previous studies have described experimental set‐ups for its breeding, which is an essential precursor for investigating the molecular and genetic mechanisms underlying its particular characteristics.

Reverse genetics approaches have been widely used in various fields of biology to directly reveal the functions of target genes involved in a broad range of biological processes. In particular, genome editing with the CRISPR/Cas system and *Tol2* transposon‐mediated transgenesis have become robust tools for genome engineering approaches. The recently established CRISPR/Cas system enables targeted genome editing with the Cas9 protein and single guide RNA (sgRNA).^16^ The Cas9 protein and the sgRNA form a ribonucleoprotein (RNP) complex and can create a double‐stranded DNA break at a target site in a manner guided by the sgRNA.^17^ The DNA break facilitates targeted gene disruption by the introduction of insertions and deletions (indels) or targeted integration of exogenous DNA fragments.^16^ An indel can result in a frameshift mutation of the coding sequence of a gene, leading to potential loss of gene function. The CRISPR/Cas system has already been applied for revealing the functions of target genes in a wide range of fish species, such as lamprey,^18^, ^19^ zebrafish,^20^, ^21^ Atlantic salmon,^22^ cichlid,^23^, ^24^ killifish,^25^, ^26^, ^27^ and medaka.^28^, ^29^, ^30^ In addition to genome editing technology, the *Tol2* transposon system is an efficient approach to establishing transgenic strains in zebrafish.^31^, ^32^ In this system, the *Tol2* transposase recognizes and excises a DNA fragment located between 5′ and 3′ *Tol2* sequences on the donor plasmid, and then randomly integrates the excised fragment into the genome.^33^ Recent studies have demonstrated that the *Tol2* system enables the efficient generation of stable transgenic strains in a wide range of fish, such as killifish,^34^, ^35^ cichlid,^36^ and stickleback.^37^

In this study, we successfully applied these genetic approaches to the rainbowfish *M. praecox* and describe here detailed procedures for genetic engineering in this species. Besides the above‐mentioned characteristics, eggs of this species do not require special treatment such as parental care, and this feature makes manipulating the eggs for genetic engineering considerably easier. To generate genetically engineered strains, we first developed a method for collecting eggs and microinjection with reference mainly to the method established in medaka.^38^ By using this procedure, we successfully demonstrated CRISPR/Cas‐mediated knockout and *Tol2* transposon‐mediated transgenesis in *M. praecox*. For the CRISPR/Cas system, we disrupted the *tyrosinase* gene, which is necessary for eumelanin synthesis in many animals, and the efficiency of gene editing in the injected embryos could be inferred from the pigmentation patterns in the body and eyes of each embryo. We also demonstrated that a *Tol2* construct, including a GFP gene driven by a ubiquitous promoter, was efficiently integrated into the genome of *M. praecox* embryos. These approaches should provide a platform for reverse genetic analysis of unique biological features of rainbowfish.

## 2 RESULTS

### 2.1 Establishing microinjection conditions for *M. praecox*

To collect fertilized eggs of *M. praecox*, a mature male and female pair was kept in a tank with a spawning mop (Figure 1C). We found that each pair usually spawned every day. Our behavioral observations indicated that they usually spawned each day within 3 h after turning on the lights. Immediately after a female accepted a male as a mate, mating occurred, after which the spawned eggs were scattered on the spawning mops. A female laid 10–20 eggs at once, which adhered to the mops via their filaments (Figure 1D). The diameter of a fertilized egg was approximately 800 μm, with long attaching filaments sprouting from the animal pole (Figure 1E–H’). As time passed after fertilization, the blastodisc swelled and the oil droplets expanded from the animal pole side to the vegetal pole side (Figure 1E–H’). Then, the first cleavage occurred approximately 90 min post‐fertilization (Figure 1G, G’) and the second cleavage occurred approximately 120 min post‐fertilization (Figure 1H, H’), at room temperature (26–28°C).

**FIGURE 2:**
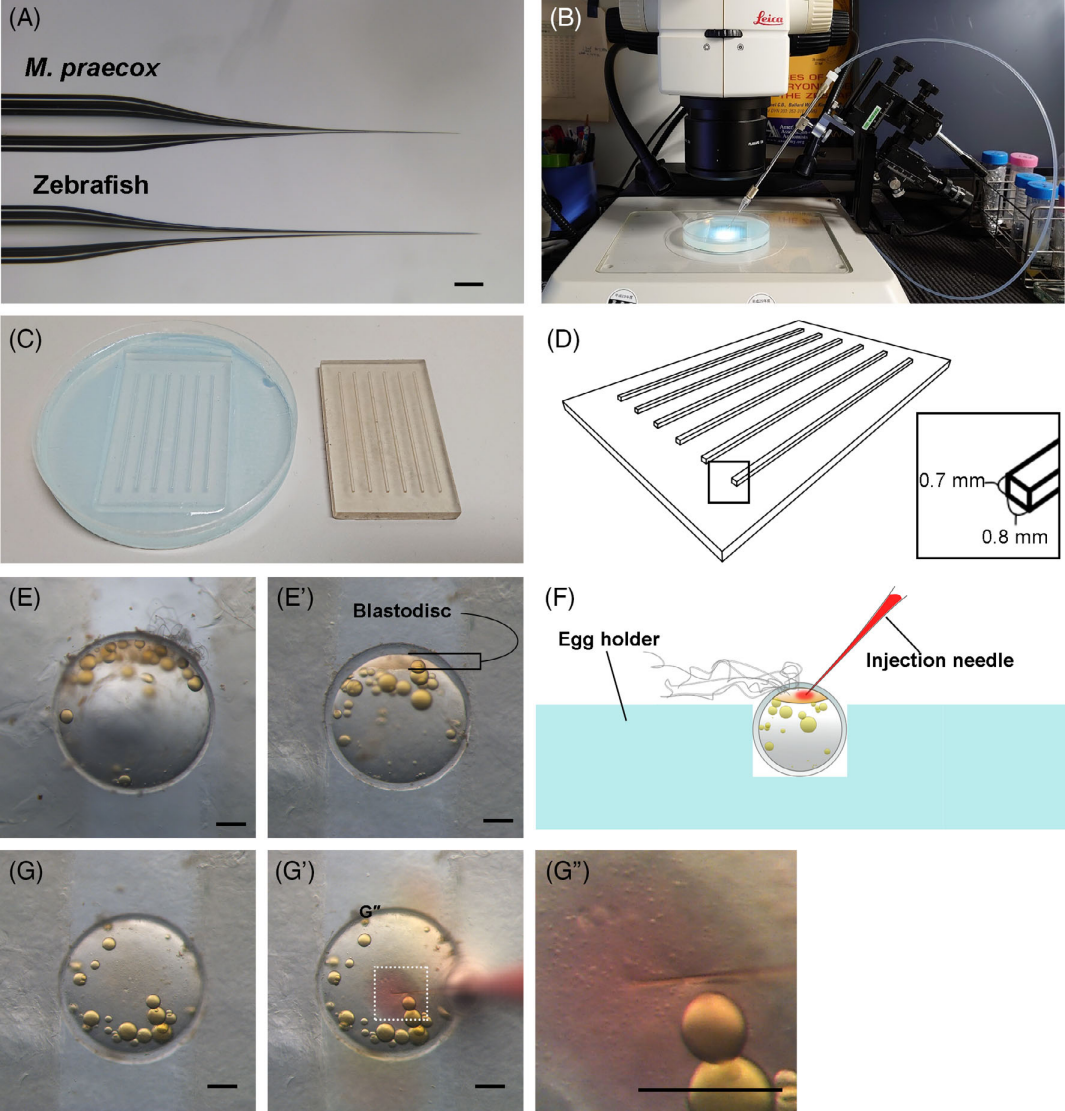
Microinjection of fertilized eggs. (A) A comparison of a microinjection needle for *Melanotaeni praecox* (top) with that for zebrafish (bottom). (B) Microinjection set‐up. Needle holder set‐up with a 3D micromanipulator to precisely control the position of the needle under a stereomicroscope. (C) A 3D‐printed mold (right) and an egg holder made of 3% agarose (left). (D) Protrusions on the 3D‐printed mold were 0.8 mm in width and 0.7 mm in height. (E), (E’) A comparison of the diameter of an *M. praecox* egg with the width of a groove in the egg holder. Images of an egg not held (E) and held (E’) in the groove. (F) A schematic illustration of the microinjection. (G)–(G”) Successful injection through the chorion to the cytoplasm was visualized by red coloration of phenol red contained in the injection solution. Scale bars indicate 200 μm.

Owing to the hard chorion of *M. praecox* eggs, injection needles optimized for zebrafish eggs cannot penetrate it. We thus optimized the injection needles to be stiffer than the zebrafish needles and sufficiently thin to avoid puncturing the blastodisc (Figure 2A). We also set up a needle holder with a three‐dimensional (3D) micromanipulator to precisely control the needle (Figure 2B). To imprint injection plates that could reliably hold the eggs during injection (Figure 2C), a 3D‐printed mold with suitably sized protrusions (0.8 mm width, 0.7 mm height) was designed to create an egg holder made of agarose (Figure 2D). We optimized the width of each groove in the egg holder, which was slightly narrower than the diameter of the *M. praecox* eggs (Figure 2E, E’). Each egg was oriented using tweezers or dissecting needle to ensure that its blastodisc, which is visible under a bright field (Figure 2E’), faced upward (Figure 2F, G). We inserted the injection needle through the chorion to the cytoplasm and applied a high pressure to inject an adequate amount of solution into the cytoplasm (Figure 2F, G”).

### 2.2 CRISPR‐mediated knockout in *M. praecox*

We first investigated whether the established method for the microinjection of *M. praecox* eggs could be applied to genome editing using the CRISPR/Cas9 system. In this study, we selected the *tyrosinase* gene, which encodes an enzyme that produces eumelanin from tyrosine,^23^, ^39^, ^40^, ^41^ as the target gene. Mutants of the *tyrosinase* gene have previously been reported in several fish species and were found to be associated with pigmentation defects early in development,^23^, ^39^, ^41^ making them suitable for evaluating the efficiency of gene disruption by phenotypic observation directly in the injected generation.

**FIGURE 3:**
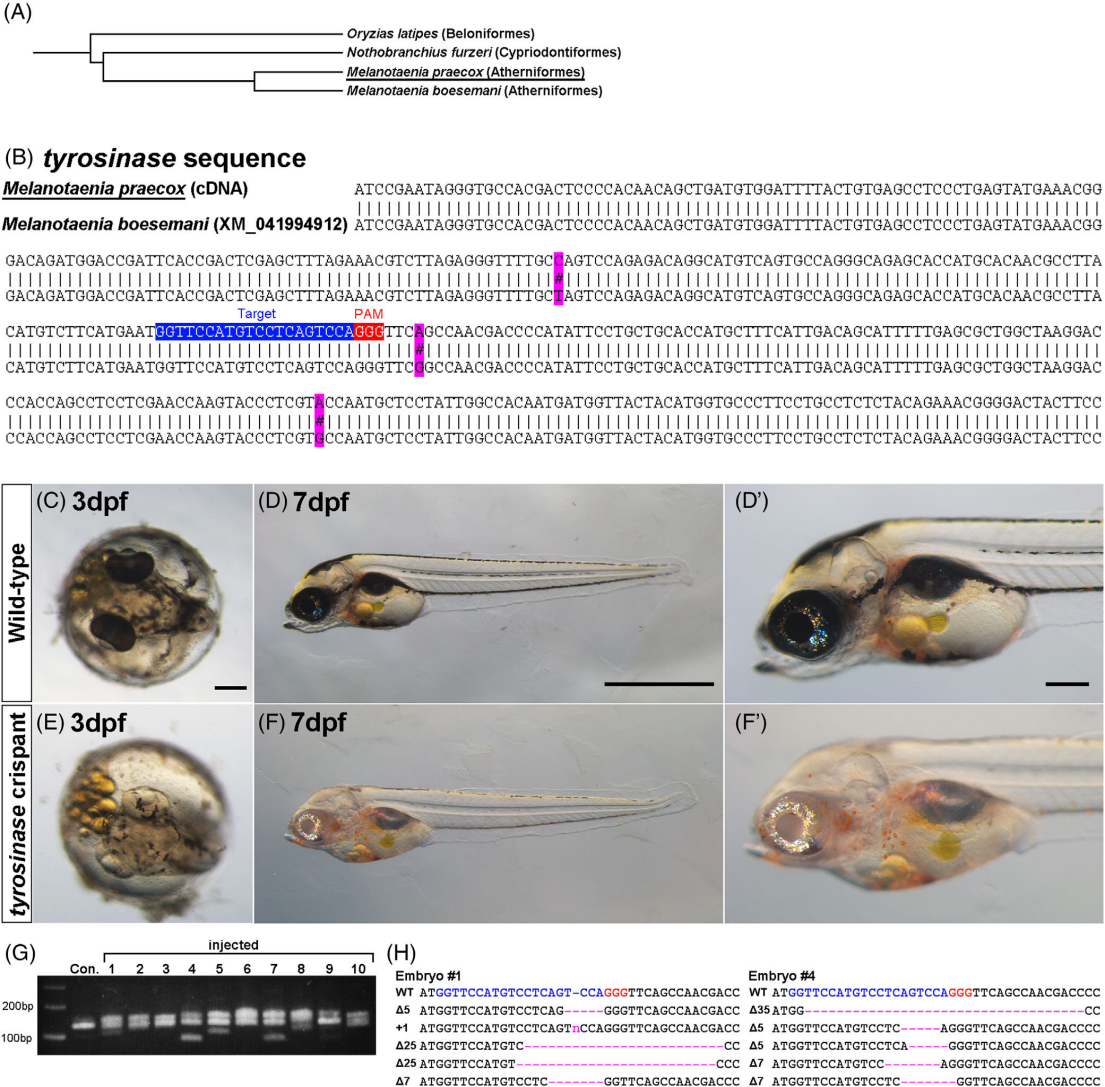
(A) A phylogenetic tree of *M. praecox* and related species.^1^, ^41^ (B) The alignment indicates differences in *tyrosinase* mRNA sequence between *M. praecox* and *M. boesemani*. The binding sequence of guide RNA (gRNA) or protospacer‐adjacent motif (PAM) is shown by a blue or red rectangle. (C)–(F’) Embryos injected with the Cas9 protein and gRNA lack black pigmentation (bottom) at 3 days post‐fertilization (dpf) (C), (E) and 7 dpf (D), (D’), (F), (F’) when the wild type shows black pigmentation (top). (G) Heteroduplex mobility assay (HMA). Multiple bands were shown in embryos injected with the Cas9 protein and the gRNA, whereas a single band was shown in the control (Con.) without injection. (H) Mutation spectrum revealed by Sanger sequencing. Magenta dashes and the letter “n” indicate identified deletions and an insertion, respectively. The gRNA target sequence and its PAM are indicated by blue and red letters, respectively. The sizes of deletions and insertions are shown to the left of each sequence (Δ, deletions; +, insertions). The numbers of the embryos (#1 and #4) in (H) correspond to the lane numbers in (G). Scale bars indicate 200 μm (C), (E), (D’), (F’) or 1 mm (D), (F).

First, we identified a partial sequence of the *M. praecox tyrosinase* gene to design sgRNAs for its targeted disruption. Primer sequences for PCR were designed with reference to the published *tyrosinase* mRNA sequence of *M. boesemani*, a species closely related to *M. praecox* that diverged approximately 35 million years ago (Figure 3A).^1^, ^42^ To obtain the *tyrosinase* sequence of *M. praecox*, we amplified the DNA fragment of the *tyrosinase* gene from cDNA using these primers and then performed Sanger sequencing. Next, we retrieved the *tyrosinase* mRNA sequence of *M. boesemani* from the NCBI website for comparison. Figure 3B shows that the partial *tyrosinase* mRNA sequences of the two species are highly conserved. We selected an sgRNA target site on the *M. praecox tyrosinase* gene. A locus homologous to this site in the *tyrosinase* gene was selected as an sgRNA target site in a previous study on cichlid (Figure 3B).^23^ We injected this sgRNA with the Cas9 protein into embryos at the one‐cell stage. At 1 day post‐fertilization (dpf), 41%–60% of the injected embryos survived in quadruplicate trials (Table 1). At 3 dpf, while the wildtype embryos without any injection developed black pigmentation (Figure 3C, D’), 30%–55% of the injected embryos survived in quadruplicate trials (Table 1), and 45%–100% of the remaining injected embryos in quadruplicate trials lacked this pigmentation, especially in the retinal pigment epithelium (Figure 3E, F’). The profiles of heteroduplex mobility assay (HMA) showed that DNA cleavage by the Cas9 RNPs resulted in indels at the sgRNA target site at the *tyrosinase* locus in the embryos lacking the black pigmentation (Figure 3G). In addition, we performed Sanger sequencing analysis of the injected embryos. The results showed that various patterns of mutations were present at the target site (Figure 3H). We speculate that various indels induced mosaicism in some cell masses in an embryo. These results suggest that RNA injection of components of the CRISPR/Cas system can efficiently induce biallelic mutations at the target site, the efficiency of which is sufficient to analyze the function of the targeted gene, even in the injected generation.

**TABLE 1:** Survival at 1 day post‐fertilization (dpf) and frequencies of positivity for mutation at 3 dpf observed in embryos injected with the Cas9 protein and a *tyrosinase* guide RNA. Results of four independent trials are shown.

	Survival rate (1 dpf)	Survival rate (3 dpf)	Positive mutation rate (3 dpf)
Trial 1	11/27 (41%)	8/27 (30%)	8/8 (100%)
Trial 2	16/29 (55%)	10/29 (34%)	6/10 (60%)
Trial 3	34/60 (57%)	29/60 (48%)	13/29 (45%)
Trial 4	12/20 (60%)	11/20 (55%)	11/11 (100%)

*Note*: Survival rate (%) = Surviving embryos (1 or 3 dpf)/Injected eggs. Positive mutation rate (%) = Positive mutants (3 dpf)/Surviving embryos (3 dpf).

### 2.3 *Tol2* transgenesis of *M. praecox*

**FIGURE 4:**
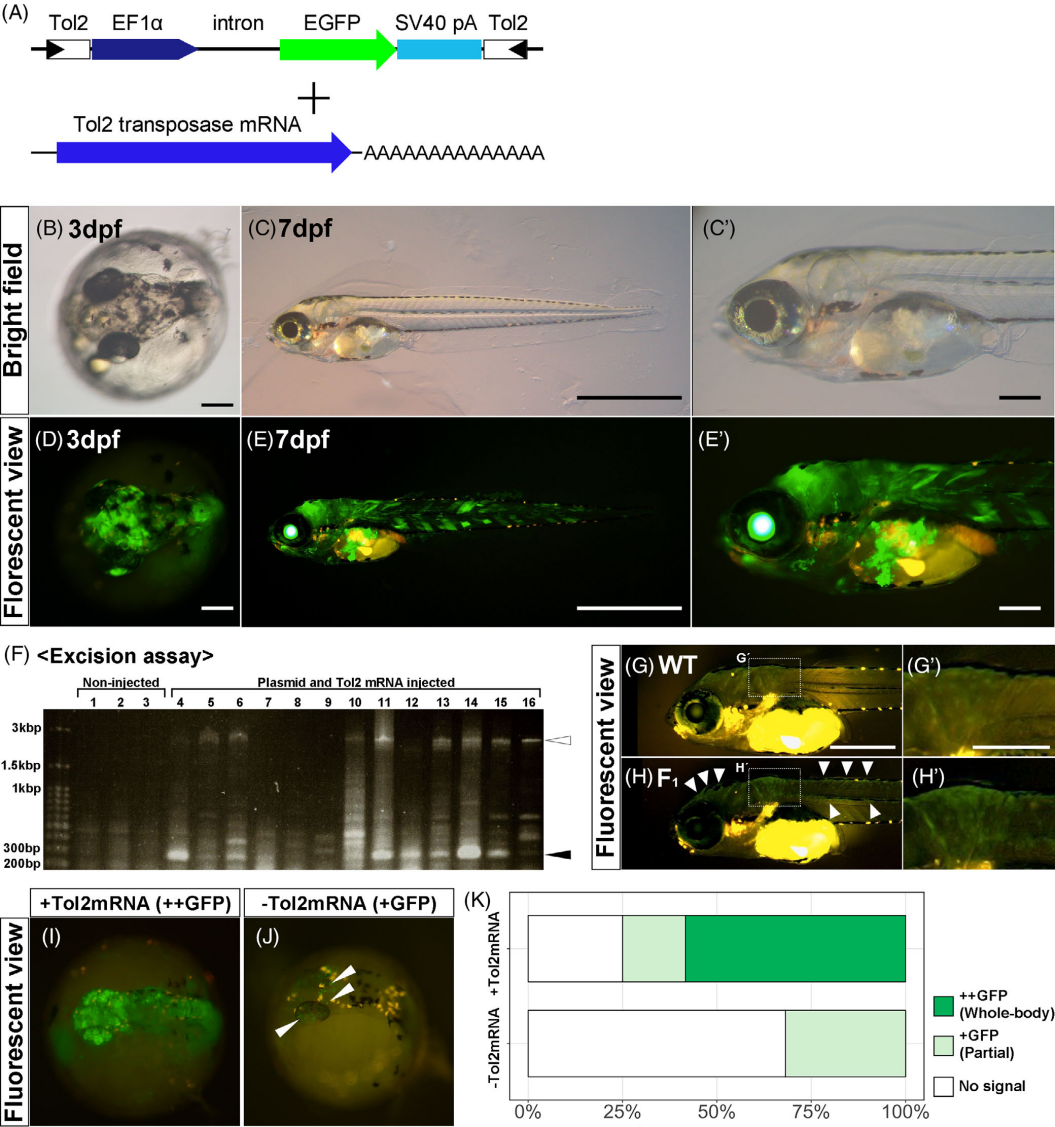
(A) Injected DNA and mRNA constructs. The *Tol2* construct (*pT2AL200R150G*) was co‐injected with *Tol2 transposase* mRNA. (B)–(E’) GFP expression in embryos injected with the *Tol2* constructs at two different developmental stages: 3 days post‐fertilization (dpf) (B), (D) and 7 dpf (C), (C’), (E), (E’). Both bright field (top) and fluorescent (bottom) images are shown. For the 7 dpf fish, magnified views are also shown (C’), (E'). (F) An electrophoresis gel image of PCR products for *Tol2* excision assay. A black arrowhead indicates the position of bands of PCR amplicons from the *Tol2* excised construct. A white arrowhead indicates the position of bands from the intact construct. (G)–(H’) Comparing the GFP expression between wildtype and F_1_ embryo at 6 dpf. White arrowheads and bracket indicate GFP‐positive signals. (I)–(K) Functional validation of the *Tol2 transposase* mRNA in terms of transgenesis efficiency. (I), (J) Representative images of GFP expression in 2 dpf embryo injected with (I) or without (J) *Tol2 transposase* mRNA. Ubiquitous GFP expression (GFP++) was observed in the embryo co‐injected with both the *Tol2* construct and the mRNA (I), while partial GFP expression (GFP+) was shown in the embryo injected only with the DNA construct (J). White arrowheads indicate GFP‐positive signals. (K) Efficiency of GFP expression with or without *Tol2 transposase* mRNA. Scale bars indicate 200 μm (B), (D), (C’), (E’), (G’), 500 μm (G) or 1 mm (C), (E).

To test whether the established injection method can be applied to transgenesis, we examined *Tol2* transposon‐mediated transgenesis in *M. praecox*. In this experiment, we used a *Tol2* construct, *pT2AL200R150G*, containing a sequence encoding EGFP under the control of the ubiquitous *Xenopus EF1α* promoter and an SV40 late polyadenylation (polyA) signal (Figure 4A).^43^ This construct can induce whole‐body GFP expression in injected embryos. We co‐injected the *Tol2* construct with *Tol2 transposase* mRNA into one‐cell‐stage embryos (Figure 4A). At 2 dpf, 24%–47% of the injected embryos survived (Table 2), and 53%–100% of them showed GFP positivity in quadruplicate trials (Figure 4B, D; Table 2). At 7 dpf, 3%–19% of the injected embryos survived (Table 2), and 78%–100% of them showed GFP positivity in septuplicate trials (Figure 4C, C’, E, E’; Table 2). Furthermore, we performed an excision assay^43^, ^44^, ^45^ of injected embryos to check whether the transposition reaction occurred (see Section 4.10 “Excision assay”). The excision assay revealed that short‐length PCR products (black arrowhead in Figure 4F), which exhibited excision of the *Tol2* fragment from the donor plasmid, were amplified in 11 of 13 embryos. In some embryos, long‐length PCR products (white arrowhead in Figure 4F), which exhibited accurate injection of the *Tol2* construct, were not amplified. Thus, the results of the excision assay showed that the *Tol2* transposition reaction usually occurred when the *Tol2* construct with *Tol2 transposase* mRNA was co‐injected into an *M. praecox* embryo. Following our observations of the injected embryos, we grew the embryos into adults and then generated F_1_ offspring to confirm germline transmission of the transgenes. Some of the F_1_ embryos, were obtained by crossing a GFP‐positive adult with a male. Comparing the GFP‐positive F_1_ embryo to the wildtype (Figure 4G, H’'), we found weak but clear GFP fluorescence in the whole body (white arrowhead in Figure 4H and white bracket in Figure 4H’). In addition, to confirm the function of *Tol2* mRNA in *M. praecox* embryos, we compared the efficiency of integration of transgenes by injection with or without *Tol2 transposase* mRNA (Figure 4I–K). Co‐injection with both the *Tol2* construct and *Tol2 transposase* mRNA resulted in whole‐body GFP fluorescence in 14 of 24 (58%) of the injected embryos and partial GFP expression in 4 of 24 (17%) of the embryos at 2 dpf (Figure 4I, K). In contrast, when we injected the *Tol2* construct without the transposase mRNA, no embryos showed whole‐body GFP fluorescence but 7 of 22 (34%) showed partial GFP fluorescence at 2 dpf (Figure 4J, K). Some orange fluorescence, seen in Figure 4J, denotes autofluorescence of the pigment cells. Statistical analysis showed that co‐injection of the *Tol2* mRNA significantly increased the frequency of embryos expressing GFP (Fisher's exact test: *p* < .0001) (Figure 4K). This suggested that the *Tol2* transposon system allows the efficient integration of transgenes in *M. praecox*.

**TABLE 2:** Survival at 2 days post‐fertilization (dpf) and frequencies of GFP expression at 2 dpf observed in embryos injected with the *Tol2* DNA construct and *Tol2 transposase* mRNA. Results of four independent trials are shown.

	Survival rate (2 dpf)	Expression rate (2 dpf)	Survival rate (7 dpf)	Expression rate (7 dpf)
Trial 1	17/36 (47%)	14/17 (53%)	—	—
Trial 2	12/31 (39%)	7/12 (58%)	—	—
Trial 3	12/33 (36%)	7/12 (58%)	—	—
Trial 4	17/39 (44%)	9/17 (53%)	—	—
Trial 5	9/38 (24%)	9/9 (100%)	2/38 (5%)	2/2 (100%)
Trial 6	12/48 (25%)	10/12 (83%)	9/48 (19%)	7/9 (78%)
Trial 7	9/36 (25%)	6/9 (67%)	1/36 (3%)	1/1 (100%)

*Note*: Survival rate (%) = Surviving embryos (2 or 7 dpf)/Injected eggs. Expression rate (%) = Expressing embryos (2 or 7 dpf)/Surviving embryos (2 or 7 dpf).

## 3 DISCUSSION

The establishment of procedures for genomic engineering has enabled investigation of the genetic mechanisms of a broad range of biological phenomena in model organisms.^20^, ^25^, ^28^, ^29^, ^30^, ^45^ In particular, genetic engineering of commonly used model fish, such as zebrafish and medaka, has provided various biological insights due to their ease of breeding and unique genetic resources.^45^, ^46^, ^47^ In this study, we established a microinjection procedure for genome editing with the CRISPR/Cas system and *Tol2*‐mediated transgenesis in *M. praecox*, which should provide the opportunity to clarify the detailed genetic mechanisms in this species.

Targeted gene disruption using the CRISPR/Cas system has enabled us to directly investigate gene functions in a broad range of organisms.^18^, ^19^, ^20^, ^21^, ^22^, ^23^, ^24^, ^25^, ^26^, ^27^, ^28^, ^29^, ^30^ In this study, we successfully demonstrated targeted mutagenesis in *M. praecox* using the CRISPR/Cas9 system on the *tyrosinase* gene as an example target gene. Because *tyrosinase* mutations cause the loss of black pigmentation in melanophores, the efficiency of gene editing in the injected embryos could be inferred from the pigmentation patterns in the body and eyes of each embryo. We found that 45%–100% of the remaining injected embryos lacked black pigmentation; this indicates that the efficiency of genome editing in *M. praecox* was sufficiently high to induce biallelic mutations in a large portion of the injected embryos (Figure 3E, F’). Therefore, the mutagenesis approach established in this study enables loss‐of‐function analysis even in the injected generation, recently referred to as “crispant” analysis. Such an analysis may have limitations, such as the occurrence of off‐target effects of CRISPR, and the phenotypes of crispants may not be congruent with stable germline mutants.^48^ Meanwhile, the survival rates of the injected embryos of *M. praecox* at 1 dpf (41%–60%) and 3 dpf (30%–55%) were lower than those of medaka or other fish species.^23^, ^28^, ^49^ A previous study with cichlids showed that optimizing the shape of the injection needle increased the survival rate of injected embryos.^23^ This result suggests that improvements to the injection needle, such as using a quartz instead of glass needle, could benefit the survival of *M. praecox* embryos and ultimately facilitate analysis of the crispants. Furthermore, a previous study on killifish demonstrated that single‐gene disruption by three different sgRNAs more efficiently induced biallelic mutations in the whole body of the injected embryo.^26^ The combination of our procedure and this approach should further facilitate the functional analysis of target genes in *M. praecox*. In addition to mutant analysis, the CRISPR/Cas system enables the generation of targeted gene knock‐in strains by inserting a donor DNA fragment into the target locus, as demonstrated in several model species: zebrafish,^21^ medaka,^30^ and killifish.^25^ Further improvements in the efficiency of genome editing with the CRISPR/Cas system may be required for the gene knock‐in experiment in *M. praecox*.

The *Tol2* transposon system has been widely used to generate stable transgenic strains in zebrafish.^31^, ^32^ Previous studies showed that this system is efficient for transgenesis in other model fish, such as killifish,^25^, ^34^ cichlid,^36^ and stickleback.^37^ In this study, we demonstrated that co‐injection of a *Tol2* DNA construct with *Tol2 transposase* mRNA induced GFP expression in >50% of the injected embryos. This efficiency of transgenesis in *M. praecox* is as high as previously described in killifish and cichlid. However, previous research on zebrafish showed that the construct that we used can also be efficiently transmitted to the F_1_ generation.^45^, ^50^ Thus, to efficiently generate a transgenic strain using the *Tol2* transposon system, we can further improve this transgenesis procedure and efficiently make transgenic fish. For example, optimization of the shape of each injection needle, *M. praecox*‐specific codon optimization of *Tol2 transposase*, and the use of highly purified in vitro‐transcribed mRNA may make the transgenesis more efficient.^45^ In this study, we obtained an F_1_ generation of GFP‐induced fish and confirmed the germline transmission (Figure 4G, H’). The intensity of GFP fluorescence was weaker in the F_1_ offspring than in the injected embryos, but whole‐body expression was shown in both. The possible reasons for this difference in GFP intensity may relate to *EF1α* promoter not firmly working the frequency of GFP‐positive embryos was reduced to 34% upon injecting the *Tol2* DNA vector without the transposase, and no embryos with whole body GFP fluorescence was observed (Figure 4K). We could not determine whether the plasmid/construct had integrated without transposase mRNA, or whether the GFP was derived from a non‐integrated plasmid/construct. Both are possible, and other possibilities cannot be excluded. Nevertheless, our data showed that co‐injection of the transposase mRNA significantly increased the frequency of embryos expressing GFP, suggesting that *Tol2* transposon‐mediated transgenesis can work efficiently in *M. praecox* embryos. Notably, *Tol2* constructs with different tissue‐specific promoters have been widely used for efficient visualization of the specific target tissues and/or cells in vivo.^51^, ^52^, ^53^ Furthermore, transgenic zebrafish for advanced genetic tools such as the Gal4‐UAS system^32^ and Cre/loxP system^53^, ^54^ have been established by *Tol2* transposon‐mediated transgenesis. These studies suggest that the *Tol2* transposon system will provide powerful tools for understanding the detailed molecular mechanisms in *M. praecox*.

To our knowledge, this is the first report of successful genomic engineering in rainbowfish and even in Atheriniformes as a whole. Taking together the features of rainbowfish and the gene editing procedure established in this study, we suggest that *M. praecox* can be used as a new model species in various fields.^10^ Because models for research in ecology, evolution, and developmental biology, rainbowfish have latent advantages; for example, the evolution of spiny rays is an interesting topic that can be investigated in rainbowfish. Although the morphology of spiny rays has diverged markedly within the Acanthomorpha lineage,^13^ the molecular basis for this has remained largely unknown because medaka, which is an experimental model of Acanthomorpha fish, has secondarily lost its spiny rays. Recent studies demonstrated the genetic engineering in several Acanthomorpha fish species with spiny rays, such as cichlid^23^, ^24^, ^36^ and clownfish^49^; however, their breeding is expensive, requiring larger spaces and the maintenance of seawater, compared with that of conventional model fish. As described in this study, the rainbowfish *M. praecox* can be kept using the same equipment as model fish and could thus become a model system to study the detailed molecular mechanisms behind the development and evolution of spiny rays. Furthermore, the rainbowfish species have significantly diversified in Australia, New Guinea, and Sulawesi. As such, these species have been studied as models in the research fields of ecology and evolution, on topics such as adaptation to lake/stream environments,^4^ adaptive radiation and sympatric speciation,^5^, ^6^ and maintenance of color morphs by sexual selection.^3^ The genetic engineering techniques will also contribute to our understanding of the molecular mechanisms behind traits involved in speciation and adaptation.

Certain features of the genetic engineering of *M. praecox* should be improved. First, the *M. praecox* genome has not yet been sequenced and reported. In this study, we cloned the *tyrosinase* cDNA sequence based on the genomic information of a related species, *M. boesemani*. Because *M. boesemani* and *M. praecox* are thought to have diverged approximately 35 million years ago,^1^ the genomic sequence of the former should differ too much to use it as a reference genome for *M. praecox*, especially in noncoding regions. Without the whole‐genome sequence, the experimental procedure would require greater time and effort than under the condition when the whole‐genome sequence is accessible, with potential limitations from unexpected outcomes, such as sgRNAs being ineffective because of unwanted mutations at potential off‐target sites. An *M. praecox* reference genome assembly will help us to conduct more detailed and accurate genetic analyses. Second, the generation time of *M. praecox* is approximately 5 months, which is still longer than those of widely used model species such as medaka, zebrafish, and killifish. Because of the longer generation time and the difficulty in obtaining and maintaining mature fish, we have only shown the germline transmission of the GFP transgene integrated by the *Tol2* transposon. Further studies on the physiological conditions for maturation may be required to establish both transgenic and mutant strains.

## 4 EXPERIMENTAL PROCEDURES

### 4.1 Animals

*M. praecox* was purchased from a pet shop (Charm Co., Ltd., Gunma, Japan). The adult fish were conventionally housed at 28°C and a pH slightly below 7.0 under a 14/10 h light/dark cycle in 1 L or 3 L tanks and were fed once or twice daily with live brine shrimp or an artificial diet. The hatched larvae were transferred to approximately 250 mL rearing tanks. The larvae were fed with live paramecium at least once every 2 days and/or brine shrimp at least once a day; the type of food depended on the size of the embryos. Elimination of dead larvae and excreta was performed as required. After the juvenile stage (approximately 50 days or standard length of >9 mm), fish were moved to larger tanks, ranging from approximately 250 mL tanks.

All experimental animal care procedures were conducted in accordance with institutional and national guidelines and regulations and were approved by Tohoku University Animal Research Committee (permit numbers: 2022LsA‐002‐02, 2020LsLMO‐018‐05). The study was carried out in compliance with the ARRIVE guidelines.

### 4.2 Molecular cloning

Partial fragments of *M. praecox* mRNAs of *tyrosinase* were obtained by PCR using a cDNA template that was reverse‐transcribed from total RNA extracted from the whole embryos at 2, 3, and 4 dpf. To amplify the fragments, we designed primers (forward: 5′‐GGT GCA AAC TGT GGT GAA TAT AGA G‐3′, reverse: 5′‐GCA GAA TCA AAC ACT TCT GGG TAA A‐3′) with reference to the mRNA sequence of *M. boesemani* (NCBI accession number No. XM_041994912.1). PCR conditions for *tyrosinase* were 2 min at 94°C; 44 cycles of 30 s at 94°C, 30 s at 57.5°C, and 1 min 50 s at 72°C; and 25 min at 72°C using TaKaRa Taq (Takara Bio). Obtained fragments were cloned using a TOPO Cloning Kit (Invitrogen) and were sequenced by Sanger sequencing. The validated sequences were used as a reference for the sgRNA probe and further PCR primer synthesis.

### 4.3 Preparation of CRISPR/Cas system

To synthesize sgRNAs, 59‐mer oligonucleotides containing a T7 promoter sequence and a 20‐mer custom target sequence were designed. The sgRNA templates were PCR‐amplified using the oligonucleotide crRNA/tracrRNA sequence (5′‐AAA AGC ACC GAC TCG GTG CCA CTT TTT CAA GTT GAT AAC GGA CTA GCC TTA TTT TAA CTT GCT ATT TCT AGC TCT AAA AC‐3′) and crRNA/tracrRNA particle (5′‐AAA AGC ACC GAC TCG GTG CC‐3′) with KOD‐Plus‐Neo (TOYOBO), and were purified using the Cica Geneus PCR & Gel Prep Kit (08111‐96; Kanto Chemical Co., Inc.). Next, sgRNAs were synthesized and purified using the CUGA7 In Vitro Transcription Kit (Nippon Genetech). Concentrations of sgRNAs were measured using a spectrophotometer (NanoDrop 2000; Thermo Fisher Scientific).

The following reagents were mixed into an injection solution: 0.25 μL of 10 mg/mL Cas9 protein (final conc. 250 ng/μL), 1 μL of 100 ng/μL sgRNA (final conc. 10 ng/μL), 1 μL of Phenol Red (#P0290, final conc. 10%; Sigma‐Aldrich), and 8.75 μL of Cas9 working buffer (20 mM HEPES; 150 mM KCl, pH 7.5, final volume 10 μL). The mixture was incubated at 37°C for 10 min to form the sgRNA–Cas9 RNP complex.

### 4.4 Preparation of *Tol2* transposon system

For the *Tol2* transposon experiment, a donor DNA plasmid (*pT2AL200R150G)* and *Tol2* mRNA were prepared in accordance with a previously described method.^45^ For the *Tol2*‐mediated system, the following reagents were mixed into an injection solution: 1 μL of 2 M KCL (final conc. 0.2 M), 1 μL of 250 ng/μL donor DNA plasmid (final conc. 25 ng/μL), 1 μL of 250 ng/μL *Tol2* mRNA (final conc. 25 ng/μL), and 7 μL of nuclease‐free water (final volume 10 μL).

### 4.5 Microinjection apparatus

The biological properties of the fertilized eggs of *M. praecox* are similar to those of medaka (*Oryzias latipes*), and therefore a microinjection procedure of *M. praecox* was established with reference to a medaka procedure.^38^ A stereomicroscope (M165C; Leica) equipped with a microinjector (FemtoJet 4i, 5252000021; Eppendorf) and a 3D micromanipulator (MN‐151; Narishige) were used for the microinjection.

Microinjection into the *M. praecox* fertilized eggs was performed in accordance with a slightly modified version of a previously established method for medaka.^38^ A 3D‐printed mold for imprinting egg holders (with 0.8 mm width and 0.7 mm height) suitable for *M. praecox* was newly created using a 3D printer (Figure 3C, D). The mold was placed on 3% Agarose S gel (312‐01193, Nippon Gene) in E3 water^55^ and then stored at 4°C until use. Microinjection needles were generated from glass capillary tubes (GD‐1; Narishige) by first using a needle puller (PN‐30; Narishige) to separate the tube into two needles and then opening the tip by gently tapping the needle on a tweezer.

### 4.6 Obtaining fertilized eggs of *M. praecox*

To obtain fertilized eggs for microinjection, a 1 L or 3 L tank containing a spawning mop was set up for each pair of mature *M. praecox* (Figure 1C). To control the onset of spawning, the male and female were separated by a transparent separator the evening before the day of injection. On the day of injection, we removed the separator and then observed the fish behavior for between 10 min and 3 h until the female started to spawn eggs. To collect the one‐cell‐stage eggs, we carefully detached the eggs from spawning mops with tweezers soon after their spawning.

### 4.7 Microinjection procedure

The chorion (egg envelope) of the *M. praecox* fertilized eggs became increasingly stiff over time, and therefore we needed to inject them soon after their collection. Collected eggs were maintained at room temperature on an egg holder filled with E3 water^55^ to prevent the embryos from drying during the injection. Each egg was inserted into grooves of the holder and oriented with tweezers or a dissecting needle by turning the cytoplasm upwards to face the microinjection needle (Figure 2F). For each microinjection needle, ~3 μL of the injection solution was backfilled using an Eppendorf Microloader (5,242,956.003; Eppendorf). A total of 2–3 nL of the solution was injected into the cytoplasm of each egg under an injection pressure at approximately 400 hPa with a constant pressure of approximately 190 hPa to avoid flow back into the microinjection needle. We continued the injection until the embryos reached the two‐cell stage, which can be easily visualized by the formation of a cell cleavage furrow at approximately 90 min post‐fertilization. Once the injection was completed, the injected embryos were gently removed from the egg holder grooves using a dissecting needle and transferred into plastic dishes with E3 water.

### 4.8 DNA extraction

Each embryo was transferred individually into a tube with genome extraction solution, 200 ng/mL Proteinase K (SRE0005; Sigma) in TE buffer, at 10 h post‐fertilization (hpf) for *Tol2* excision assay or 3 dpf for CRISPR/Cas9 mutation analysis. Each embryo was lysed by incubation at 50°C overnight, and then the residual proteinase activity was inactivated by incubation at 95°C for 15 min.

### 4.9 Mutation analysis

Mutations at the sgRNA target site on the *tyrosinase* gene in the injected embryos were evaluated using a HMA with primers (forward: 5′‐AGT CCA GAG ACA GGC ATG TCA GTG C‐3′, reverse: 5′‐ATG AAA GCA TGG TGC AGC AGG AAT ATG GGG‐3′). The PCR conditions were as follows: 2 min at 94°C and then 35 cycles of 15 s at 94°C, 30 s at 65°C, and 10 s at 68°C using KOD‐Plus‐Neo (TOYOBO) with the genomic DNA of the 3 dpf embryos as a template.

For sequence analysis at the target site, the genomic region, including the target site of sgRNAs, was amplified with primers (forward: 5′‐CCA CTT GCT ATT TTC CCA TCC CTT TGC C‐3′, reverse: 5′‐CCA CAT TTA CTC ATT ACT GTC TCC TGC‐3′). The PCR conditions were 2 min at 94°C and then 40 cycles of 15 s at 94°C, 30 s at 66°C, and 20 s at 68°C using KOD‐Plus‐Neo (TOYOBO). The PCR products were purified with Cica Geneus PCR & Gel Prep Kit (08111‐96; Kanto Chemical Co., Inc.). Each sequence chromatogram obtained by direct Sanger sequencing of the PCR products was analyzed using the Synthego ICE CRISPR analysis tool (https://ice.synthego.com/)^24^ to infer the mutation spectrum in each injected embryo.

### 4.10 Excision assay

To evaluate the excision activity by the *Tol2* transposase in the injected embryos, the transient excision assay was performed as described previously^43^, ^45^ with some modifications. Using the genomic DNA extracted from 10 hpf embryos as a template, the excision product was amplified using Takara Taq (Takara Bio), and the primers exL3 (5′‐CCA AGC GCG CAA TTA ACC CTC ACT‐3′) and exR3 (5′‐GCC TCT TCG CTA TTA CGC CAG CT‐3′).

### 4.11 Observation of larvae

Embryos injected with or without Cas9 RNA were observed under a bright field of a stereomicroscope (M165 C; Leica) with a digital camera (MC170 HD; Leica). Embryos injected with the *Tol2* construct and/or the *Tol2* transposase mRNA were observed under a fluorescent stereomicroscope (M165 FC; Leica) with a digital camera (DP74; Olympus). After hatching, the larvae were anesthetized with 0.025% MS222/E3 and placed on 1% agarose gel/E3. The larvae were immediately transferred to a small case filled with system water and sprayed water to larva to awaken.

### 4.12 Statistical analysis

A percentage stacked bar chart showing the frequencies of GFP‐positive embryos was generated with the R (https://www.r-project.org/) package ggplot2. For quantitative analysis of the frequencies of GFP‐positive embryos, Fisher's exact test was performed in R using the fisher.test function.

## FUNDING INFORMATION

This study was supported by JSPS KAKENHI (Grant Numbers 22K06232, 20H04854, 22H02627, 21K19202, and 21H05768) and Takeda Science Fundation (Grant Number 2022036015).

## CONFLICT OF INTEREST STATEMENT

The authors declare no conflict of interest.

## Data Availability

All raw data and imaging files are available upon request from the authors.
